# Cholesteatoma: Canalplasty for External Auditory Stenosis in a Pediatric Patient

**DOI:** 10.7759/cureus.51188

**Published:** 2023-12-27

**Authors:** Amir Elzomor, Marissa Firlie, Nicklas Orobello, Jonathan Murnick, Brian K Reilly

**Affiliations:** 1 Division of Otolaryngology, Head and Neck Surgery, The George Washington University School of Medicine, Washington, USA; 2 Department of Pediatric Otolaryngology, Children's National Medical Center, Washington, USA; 3 Department of Pediatric Radiology, Children's National Medical Center, Washington, USA

**Keywords:** hearing loss, hemifacial hypertrophy, pediatric otolaryngology, canalplasty, external auditory canal cholesteatoma

## Abstract

External auditory canal (EAC) stenosis is the narrowing of the external auditory meatus to less than 4 mm. Severe stenosis of the EAC may inhibit the ability to conduct sound and may lead to the formation of a cholesteatoma. While most cases of EAC stenosis may be managed nonoperatively, the significant impact that the associated symptoms can have on patients may require surgical intervention. Progression of the cholesteatoma can erode the bony ossicles, may encase the facial nerve, and impact infection risk causing chronic otorrhea, and further worsening patient quality of life. We present the case of a pediatric patient who presented due to chronic left-sided hearing loss. Further examination and imaging demonstrated near-total obstruction of the left EAC secondary to a soft tissue mass and evidence of bony hypertrophy. Following a canalplasty, the patient now has returned to baseline hearing and has no associated complications. Canalplasty remains a safe, effective surgical intervention for EAC stenosis complicated by cholesteatoma.

## Introduction

External auditory canal (EAC) stenosis is relatively uncommon and is defined as the narrowing of the external auditory meatus to less than 4 mm [1]. It may be congenital or the result of numerous acquired causes, including trauma and infection. This may inhibit the ability to conduct sound as well as clearing of squamous debris. While most cases of EAC stenosis may be managed nonoperatively, the inability to conduct sound or clear squamous debris may predispose patients to developing conductive hearing loss, recurrent otitis media, or formation of a cholesteatoma. These patients may require surgical intervention due to the significant impact these complications may have on quality of life. Surgical techniques for canalplasty have been previously described and are outside the scope of the present manuscript. Preoperative workup of EAC stenosis with hearing loss includes microscopic exam, formal audiological testing, and computer tomography (CT) scans [2]. Canalplasty remains a safe and effective surgical intervention for EAC stenosis with the development of cholesteatoma. We present the case of a pediatric patient who successfully underwent canalplasty for left-sided EAC stenosis complicated by cholesteatoma formation.

## Case presentation

A 13-year-old male with a history of neurocutaneous nodules and hypothyroidism presented to the otolaryngology clinic for the management of chronic left-sided hearing loss. He had no associated symptoms (e.g., otorrhea, otalgia, vertigo) and no history of recurrent ear infections. Microscopic examination of the left ear revealed soft tissue obstruction of the EAC such that the tympanic membrane (TM) could not be visualized.

The patient had been receiving serial computed tomography (CT) scans since the age of four to monitor the progression of hemifacial hypertrophy. As the patient’s ear and associated structures continued to expand, the EAC became obstructed. He was followed for several years in the craniofacial clinic before presenting to the otolaryngology clinic for progressive left-sided hearing loss.

**Figure 1:**
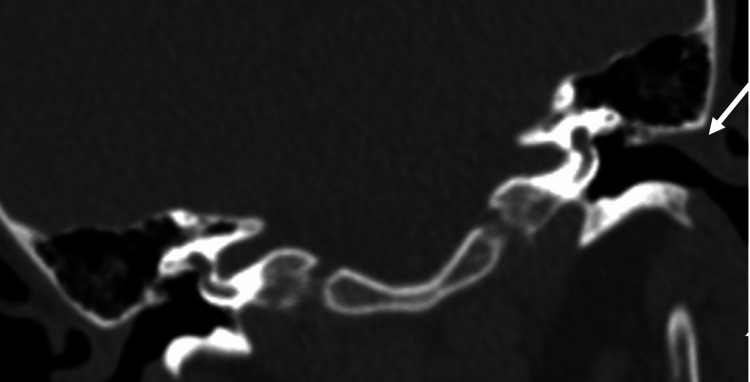
Coronal cut of high-resolution computed tomography (CT) scans through the temporal bones when the patient was age four. Soft tissue obstruction of the left external auditory canal (EAC) is noted (arrow).

**Figure 2:**
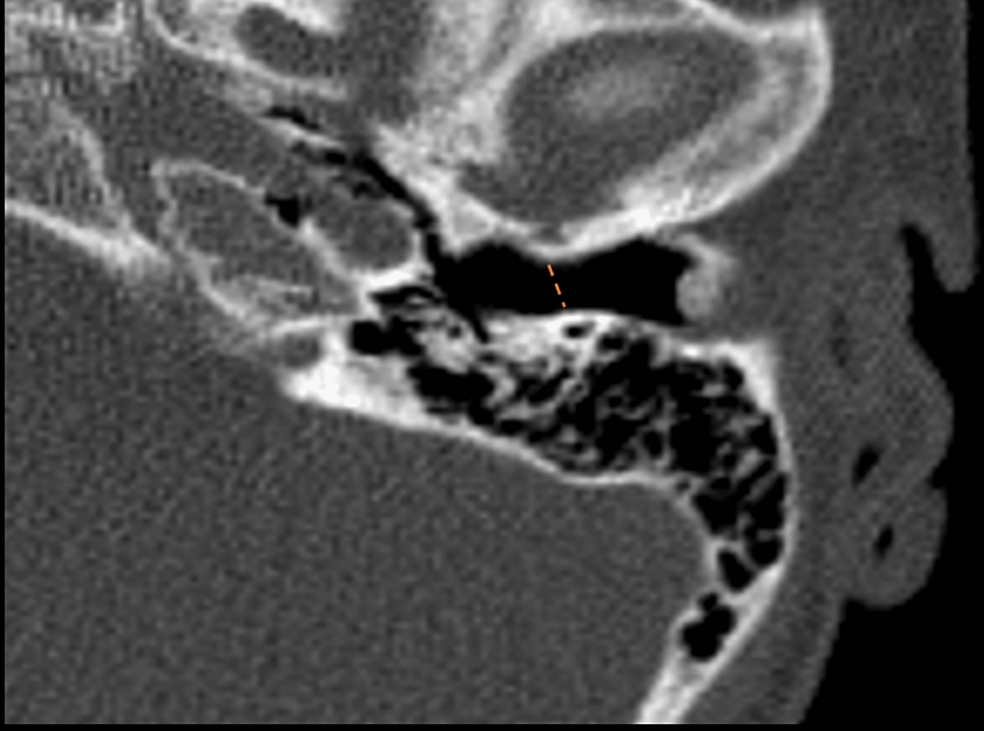
Axial cut of CT scans through the left temporal bone when the patient was age ten. Bony hypertrophy of the left external auditory canal (EAC) with lateral soft tissue obstruction and further canal stenosis is noted (dotted line).

**Figure 3:**
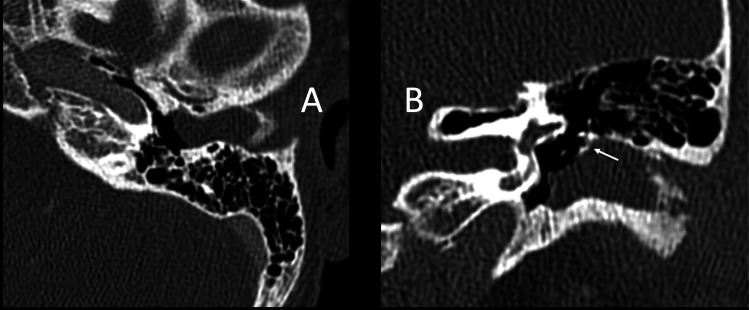
Axial cut (A) and coronal cut (B) of CT scans through the left temporal bone when the patient presented at age 13. Bony hypertrophy and soft tissue obstruction of the left external auditory canal (EAC) are again noted. Cholesteatoma with scutal erosion is noted on the coronal cut (arrow).

A soft tissue mass was demonstrated on the CT scan of the temporal bones with intravenous (IV) contrast beginning at the age of four (Figure 1). Subsequently, at the age of ten, expansile changes of the bony EAC were also noted, with evidence of bony hypertrophy extending into the EAC and further contributing to canal stenosis (Figure 2). The most recent imaging was obtained when the patient presented to our clinic at the age of 13. The CT scan demonstrated canal skin and debris trapped within the EAC and the progression of the cholesteatoma with evidence of bony erosion of the scutum (Figure 3). Audiometric testing was notable for a moderate conductive hearing loss of the left ear, with air-bone gaps (ABGs) of 30-40 dB. This was associated with a flat, type B, tympanogram with a small canal volume.

**Figure 4:**
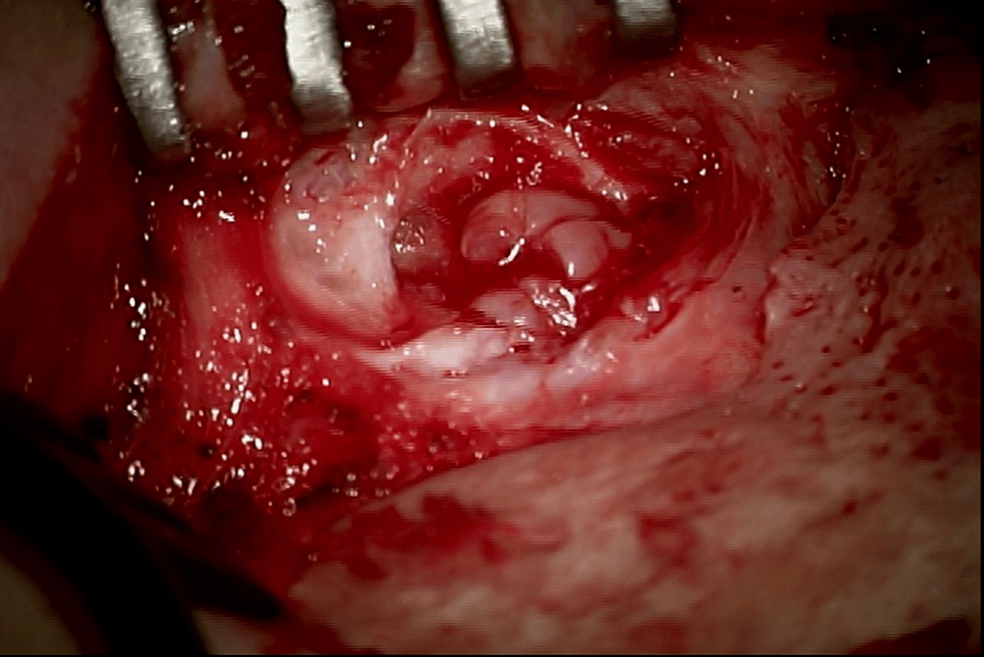
Intraoperative image demonstrating cholesteatoma contributing to the external auditory canal (EAC) stenosis

**Figure 5:**
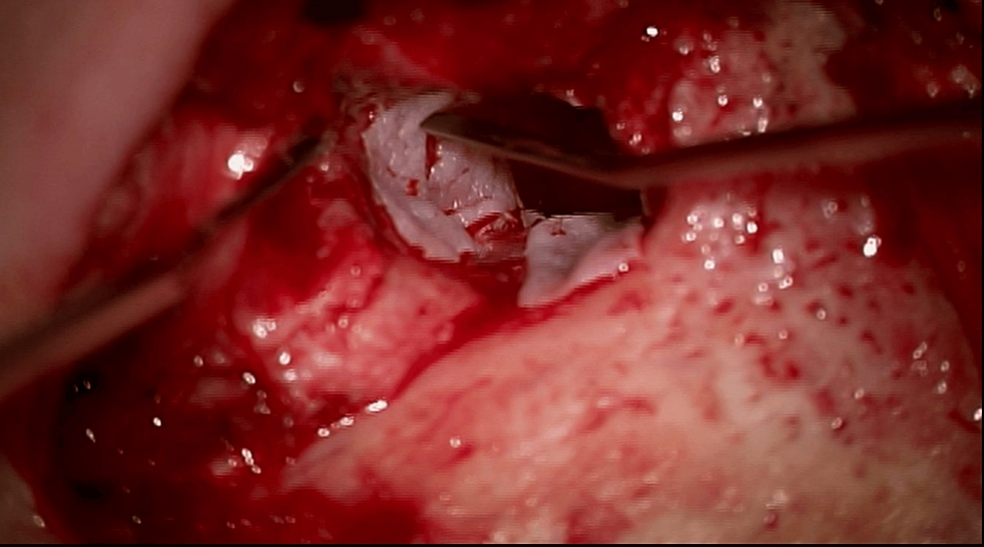
Intraoperative image demonstrating canalplasty and split-thickness skin graft (STSG) being placed in the external auditory canal (EAC)

The patient was taken to the operating room for canalplasty with excision of the soft tissue EAC mass via a postauricular approach (Figure 4). There was hypertrophic bone along the lateral extent of the bony EAC that obstructed the canal. A canalplasty was performed, and the hypertrophic bone was drilled. Cholesteatoma with associated diseased skin was also removed and sent to pathology as a permanent specimen. Canalplasty is a surgical procedure performed in order to widen a narrowed external auditory meatus. In this patient, canalplasty was performed such that the tympanic membrane could be visualized and the canal could accommodate a size #4 otologic speculum. The tympanic membrane was intact - given this and the absence of middle ear disease on the CT scan, a tympanotomy was not performed. As such, attention was turned to reconstruction. The extent of canal cholesteatoma necessitated the reconstruction of the canal with a split-thickness skin graft (STSG) (Figure 5). A STSG was harvested in the usual fashion, and thin strips of skin were placed along the bony EAC, laid from medial to lateral, in a radial fashion. The canal was then gently packed with Ofloxacin-soaked Gelfoam® and Bacitracin ointment to facilitate the healing of the skin grafts.

The patient was seen after surgery at one and four months postoperatively. At the time of his most recent follow-up appointment, his EAC was widely patent and STSGs had healed appropriately. Audiometric testing revealed normal left-sided air conduction thresholds across all frequencies, with a pure tone average (PTA) of 2 dB. The tympanogram was normal.

## Discussion

The external auditory canal (EAC) originates from the ectoderm of the first branchial groove [3]. Typically, the external auditory meatus is formed as the first branchial groove deepens to form a funnel-shaped tube, extending medially, during the eighth week of embryonic development. This forms the lateral, fibrocartilaginous 1/3 of the EAC. Medially, a meatal plate extends from the primary external auditory meatus to the tympanic membrane (TM). This meatal plate then canalizes during the twenty-first week of development to form the medial 2/3 of the EAC. Thus, malformation of the first branchial arch or failure of resorption of the mesenchymal meatal plate can both lead to congenital aural atresia or congenital EAC stenosis [3].

External auditory canal stenosis is defined by a canal diameter of < 4mm [1]. Although most patients with EAC stenosis can be managed nonoperatively [4], stenotic EACs are limited in their ability to conduct sound and to allow clearance of squamous debris. Patients with impaired sound conduction and canal clearance can suffer from conductive hearing loss, recurrent otitis externa, and/or cholesteatoma. As such, some patients will necessitate surgical intervention to address the aforementioned complications [4].

The surgical objectives of canalplasty for EAC stenosis are both to improve conductive hearing and to facilitate the clearance of squamous and ceruminous debris from the EAC. Failure of the canal to clear squamous debris can lead to canal cholesteatoma, which is common in cases of EAC stenosis with a prevalence of 43% demonstrated in a recent systematic review [5]. Some reports estimate the prevalence in cases of canal stenosis to be as high as 91% [1,5,6]. Thus, clinicians should maintain a high index of suspicion in patients with EAC stenosis, as cholesteatoma progression can erode the ossicles, facial nerve, and additional local structures to further worsen patient quality of life. Specifically, patients with a bony canal opening of < 2mm are at an especially high risk for the formation of cholesteatoma [1] and surgical intervention should be strongly considered.

The preoperative work-up of patients considered for canalplasty is similar to that obtained for patients with other otologic pathology. Namely, binocular microscopic examination should be performed, followed by formal audiometric testing and CT scans. Diffusion weighted-magnetic resonance imaging (DW-MRI) should be considered in unclear cases, as non-echo planar (EPI) DW-MRI imaging can provide improved spatial resolution with thinner slice width than other imaging modalities [7]. Patients with a significant conductive hearing loss and objective evidence of EAC stenosis should be considered for canalplasty, especially in cases suspicious for cholesteatoma.

Canalplasty is often performed via a postauricular approach, as presented herein. The reconstructive techniques used in cases of EAC stenosis are variable and dependent on the extent of canal widening performed. For example, skin grafting with STSGs is often employed to minimize bone exposure and facilitate re-epithelialization of the canal. Skin grafts provide an epithelial framework on which newly generated epithelial cells can migrate to establish an appropriately epithelialized canal [8]. It is essential that the skin grafts are thin enough to allow for graft uptake [9].

This is analogous to the epithelial “bridge” that migrates to the center of a tympanic membrane perforation in the initial phases of perforation healing [10]. Conversely, the exposed bone may provide a nidus for infection, granulation tissue, and scar formation, placing the patient at risk for subsequent re-stenosis [11]. Indeed, re-stenosis is one of the most dreaded and most common postoperative complications of canalplasty, with a prevalence as high as 20% reported in the literature [8,12-15].

Similarly, various canal packing mechanisms have been proposed to reduce the risk of postoperative re-stenosis. Long-term stenting with earmolds and hearing aids was found to significantly reduce the relative risk of postoperative stenosis in a retrospective review of 96 patients undergoing canalplasty over an extended period [16]. Merocel® wicks [8,11] and packing strip gauze can also be used to promote healing and - in cases of skin graft use - facilitate graft uptake. In the present case, ofloxacin-soaked Gelfoam® was used so as to avoid dislodging of the separate skin grafts as well as to negate the need for subsequent packing removal.

## Conclusions

While the majority of patients with EAC stenosis may be managed nonsurgically, the formation of a cholesteatoma may lead to numerous complications. Decreased sound conduction and decreased ability to remove debris may significantly reduce patient quality of life and may necessitate surgical intervention. Canalplasty remains a safe and effective technique for the management of EAC stenosis. Postoperative re-stenosis remains the most common complication after a canalplasty and this risk may be mitigated by ensuring that the skin graft is thin enough. EAC packing after a canalplasty is also an effective measure to help reduce the risk of re-stenosis.

## References

[1] Cole RR, Jahrsdoerfer RA (1990). The risk of cholesteatoma in congenital aural stenosis. Laryngoscope.

[2] Gassner EM, Mallouhi A, Jaschke WR (2004). Preoperative evaluation of external auditory canal atresia on high-resolution CT. AJR Am J Roentgenol.

[3] Bluestone C, Stool S, Arjona S (1983). Bluestone and Stool Pediatric Otolaryngology, 1st ed. Bluestone and Stool Pediatric Otolaryngology, 1st ed.

[4] Sanna M, Russo A, Khrais T, Jain Y, Augurio AM (2004). Canalplasty for severe external auditory meatus exostoses. J Laryngol Otol.

[5] Chan CY, Karmali SA, Arulanandam B, Nguyen LH, Duval M (2023). Cholesteatoma in congenital aural atresia and external auditory canal stenosis: a systematic review. Otolaryngol Head Neck Surg.

[6] Casale G, Nicholas BD, Kesser BW (2014). Acquired ear canal cholesteatoma in congenital aural atresia/stenosis. Otol Neurotol.

[7] Muzaffar J, Metcalfe C, Colley S, Coulson C (2017). Diffusion-weighted magnetic resonance imaging for residual and recurrent cholesteatoma: a systematic review and meta-analysis. Clin Otolaryngol.

[8] Teufert KB, De la Cruz A (2004). Advances in congenital aural atresia surgery: effects on outcome. Otolaryngol Head Neck Surg.

[9] Haidar YM, Walia S, Sahyouni R, Ghavami Y, Lin HW, Djalilian HR (2016). Auricular split-thickness skin graft for ear canal coverage. Otolaryngol Head Neck Surg.

[10] Johnson AP, Smallman LA, Kent SE (1990). The mechanism of healing of tympanic membrane perforations. A two-dimensional histological study in guinea pigs. Acta Otolaryngol.

[11] Windsor AM, Ruiz R, O'Reilly RC (2020). Congenital soft tissue stenosis of the external auditory canal with canal cholesteatoma: case report and literature review. Int J Pediatr Otorhinolaryngol.

[12] Chang SO, Lee JH, Choi BY, Song JJ (2007). Long term results of postoperative canal stenosis in congenital aural atresia surgery. Acta Otolaryngol Suppl.

[13] Moss WJ, Lin HW, Cueva RA (2016). Surgical and audiometric outcomes for repair of congenital aural atresia and hypoplasia. JAMA Otolaryngol Head Neck Surg.

[14] Digoy GP, Cueva RA (2007). Congenital aural atresia: review of short- and long-term surgical results. Otol Neurotol.

[15] Lavy J, Fagan P (2000). Chronic stenosing external otitis/postinflammatory acquired atresia: a review. Clin Otolaryngol Allied Sci.

[16] Moon IJ, Cho YS, Park J, Chung WH, Hong SH, Chang SO (2012). Long-term stent use can prevent postoperative canal stenosis in patients with congenital aural atresia. Otolaryngol Head Neck Surg.

